# Impact of Urbanization and Socioeconomic Factors on the Distribution of Cutaneous Leishmaniasis in the Center of Morocco

**DOI:** 10.1155/2020/2196418

**Published:** 2020-01-03

**Authors:** H. El Omari, A. Chahlaoui, F. Talbi, K. Ouarrak, A. El Ouali Lalami

**Affiliations:** ^1^Natural Resources Management and Development Team, Laboratory of Health and Environment, Faculty of Sciences, Moulay Ismail University, Meknes, Morocco; ^2^Laboratory Biotechnology and Preservation of Natural Resources, Faculty of Sciences Dhar El Mahraz, Sidi Mohamed Ben Abdellah University, 30000 Fez, Morocco; ^3^Higher Institute of Nursing Professions and Health Techniques Fez, Regional Health Directorate, El Ghassani Hospital, Fez, Morocco

## Abstract

**Background:**

Parasitic diseases, in particular leishmaniasis, are still a public health problem in several countries and in Morocco.

**Methods:**

The data used are those of epidemiological surveillance collected in the registers of the prefectural epidemiology cell (PEC); however, the socioeconomic data were obtained from the High Commissioner for Planning. The Pearson correlation test was used to determine the correlation between the different variables.

**Results:**

In total, 70 cases were recorded by the prefectural epidemiology cell (PEC) during the period from 2009 to 2015. 46% of the cases come from rural areas while 54% of the cases come from urban areas. The Pearson test shows the existence of a significant relationship between the number of cases recorded and the type of environment (*r* = 0.49, *p* value = 0.02), and population rate (*R* = 0.849 and *p* ≤ 0.001). However, in our case, the poverty rate does not influence CL's distribution.

**Conclusion:**

Our results show that the CL affects the majority of the municipalities with predominance of the urban environment, so the distribution of cases of this pathology is not influenced by the poverty; however, the urbanization and the number of inhabitants have a positive impact on the distribution of this scourge.

## 1. Introduction

Leishmaniases are parasitic diseases common to humans and certain animals (anthropozoonoses) [1]. They are caused by flagellate protozoa of the genus *Leishmania* and transmitted by the bite of an insect vector, the female hematophagous sandfly [2, 3].

Those are chronic diseases, difficult to treat, evolving over several months in endemic areas [4]. According to recent reports, Leishmaniasis is endemic in 98 countries, and around 700000 to 1 million new cases and some 26 000 to 65 000 deaths occur annually. [5].

They are often subject to a recrudescence following the modification of natural environments, mainly due to climatic changes and to anthropogenic factors that favor their geographic extension to unaffected areas [6].

As is the case in many Mediterranean countries, Morocco is an endemic country of the cutaneous leishmaniasis (CL); leishmaniasis is endemic in some regions and presents a real health problem [7]. Indeed, in 2015, 2813 have been reported among with an incidence of 7.9 per 100,000 inhabitants. The infection occurs in three forms: the zoonotic CL at Leishmania major in the south; the reservoir of which is a rodent is transmitted by the vector *P. papatasi*, anthroponotic CL by Leishmania tropica at the center with emergence of new 50 outbreaks in the north; this type is transmitted by *P. sergenti* and *P. Chabaudi* and is maintained by the human and dog reservoir. CL is caused by *Leishmania infantum* in the north whose reservoir is the dog, and the offending vectors are *P. longicuspis*, *P. perniciosus*, and *P. ariasi*. [8, 9].

The geographical distribution of each species is dependent on the distribution of these vectors, which is correlated with bioclimatic [10], and topographic conditions, and also with HIV confections [11].

The control and surveillance of this epidemic in Morocco is based on case detection, and treatment cases for anthroponotic leishmaniasis, the fight against reservoirs (rodents) for zoonotic leishmaniasis, but mainly on vector control using insecticides and improving hygienic conditions.

Because CL à *L.tropica* is endemic in the region of Meknes (Center of Morocco), the present work aims to diagnose the epidemiological situation of CL in the city of Meknes in central Morocco during the years 2009–2015, by analyzing the impact of urbanization and socioeconomic factors on the distribution of this scourge.

## 2. Material and Methods

### 2.1. Study Area

**Figure 1:**
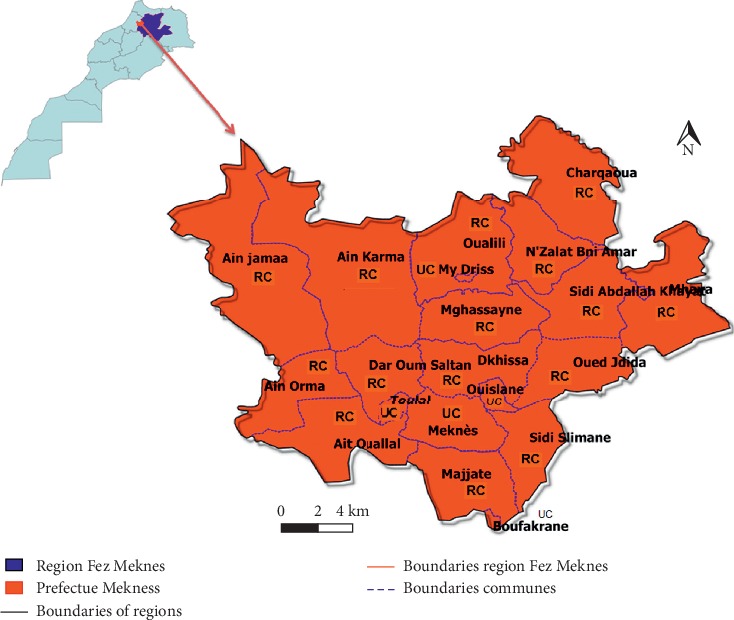
Location of the Meknes prefecture in the center of Morocco. UC, urban commune; RC, rural commune.

The prefecture of Meknes is a predominantly urban subdivision of the Fez-Meknes region in central Morocco, which extends over an area of about 1786 square kilometers. Its prefectural territory is divided into 20 communes, including 15 rural communes (Figure 1).

The prefecture of Meknes occupies a strategic geographical position, thanks to the positioning of the city of Meknes, at the crossroads of two main roads of the Kingdom of Morocco (national and provincial roads, sections of highways, and railroads connecting Marrakech and Oujda), also between two sets of mountains: the Pre-Rif and the Western Middle Atlas.

The legal population of the predominantly urban prefecture reached 835695 inhabitants in 2014 [12].

### 2.2. Acquisition of Data

The exploitable health data for this study are extracted from the weekly files for the surveillance and follow-up of leishmaniasis cases. In 2009–2015, a total of 70 leishmaniasis cases were the subject of an epidemiological study. Data on human cases were obtained from the Prefectural Epidemiology Unit (PEU) of the city of Meknes.

These socioeconomic data, namely, poverty rate, popular density, and type of environment (urban/rural) were obtained from the High Commissioner for Planning [12].

### 2.3. Data Processing

To test the null hypothesis of the absence of linear relationship between the variables, we used the Pearson correlation test to determine the absence or presence of a significant linear relationship between variables.

The calculation of the Pearson correlation coefficient is based on the calculation of the covariance between two continuous variables. For the correlation coefficient to be significant, the value of *p* must be smaller than 0.05 (*p* value <0.05). The calculations of the correlation coefficient and the *p* value are carried out using the IBM SPSS Statistical software version 24.0.

For spatial representation of the relationship between socioeconomic factors, urbanization, and CL, we used the QGIS 2.18 software, it is designed to collect, store, process, analyze, manage, and present spatial and geographic data. Using this tool, we produced thematic maps (risk map) containing health and socioeconomic data, which facilitates the interpretation of the results.

## 3. Results

This study presents an analysis of the epidemiological profile of cutaneous leishmaniasis and the impact of urbanization and socioeconomic factors on the distribution of this epidemic in the prefecture of Meknes.

To reach this object, we are interested in the retrospective study of CL cases in this region during the period 2009–2015.

### 3.1. Temporal Evolution of CL (2009–2015)

**Figure 2:**
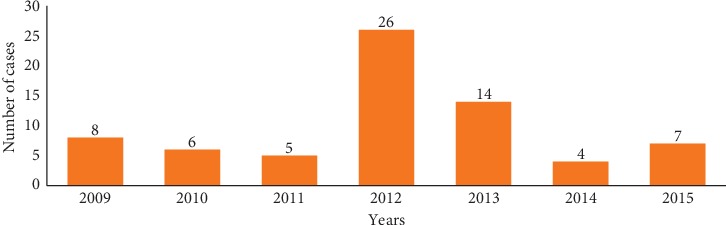
Temporal evolution of cases of cutaneous leishmaniasis (2009–1015).

The evolution of the annual number of leishmaniasis cases, reported during the period 2009–2015 in the region of Meknes, is illustrated in the Figure 2.

The temporal profile is characterized by the endemic-epidemic character that results in the permanent existence of the disease with rapid periods of propagation such as the case of the year 2012. Indeed we note that, between 2009 and 2011, the number of registered cases is gradually decreasing while the annual number of new cases of CL has increased by 20 cases between 2011 and 2012. In addition, there is a significant and gradual regression of annual enrollment in 2013.

The statistical study of these results shows that there is no relation between the distribution of cases and time (*p* value is greater than 0.05).

### 3.2. Impact of Urbanization and Socioeconomic Factors on the Distribution of CL

#### 3.2.1. Urbanization

**Figure 3:**
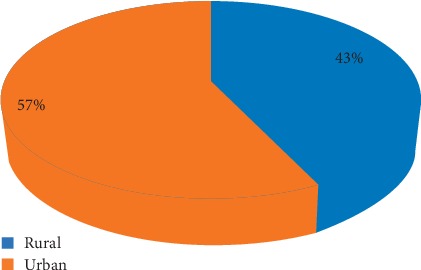
Distribution of cases of CL by medium.

Our study area consists of 20 communes, according to the study of the distribution of cases of CL; we find that CL is relatively more common in urban areas with 57% against 43% in rural areas (Figure 3).

Statistical analysis by the Pearson test shows the existence of a significant relationship between the number of cases recorded and the type of environment (*r* = 0.49, *p* value = 0.02).

#### 3.2.2. Poverty

**Figure 4:**
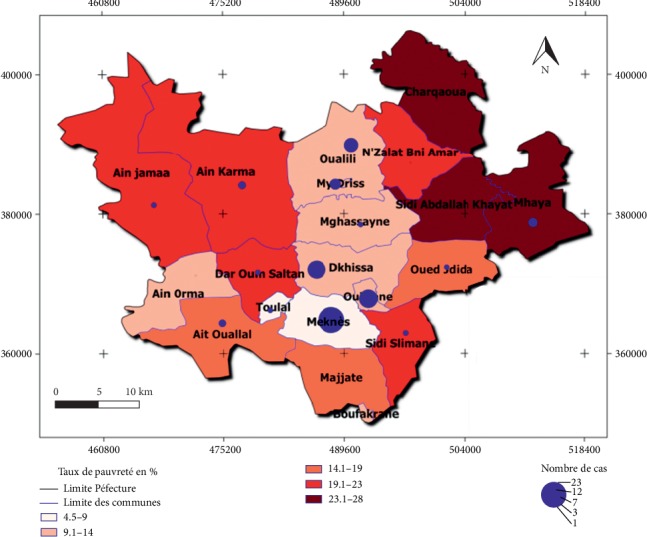
Distribution number of cases of CL and the poverty rate by municipalities.

At the Meknes Prefecture, the poverty rate varies from 4.5% to 28%. The superposition of epidemiological data (Number of cases) and those of the poverty rate (Figure 4) show that there is no link between the distribution of poverty and the distribution of cases in the communes.

In fact, municipalities that have recorded a significant number of cases of CL during this period of study are characterized by a low rate of poverty. In the urban commune of Meknes where 23 cases were noted from 2009 to 2015, the poverty rate is around 4.5%. At the same time, Dkhissa and Ouislane communes, which recorded 11 and 12 cases, respectively, have a poverty rate of around 15.6% and 11.2%. Also, Ain Karma, Ait Ouallal, and Toulal communes with a poverty rate of 19.75%, 16.23%, and 7.07% recorded a number of cases of 2, 2, and 1, respectively.

The statistical analysis showed the negative correlation between the poverty rate of the communes and the number of cases of CL in Meknes (*R* = −0.544 and *p* value = 0.013). Therefore, the poverty rate of municipalities has no influence on the distribution of the epidemic.

#### 3.2.3. Population

**Figure 5:**
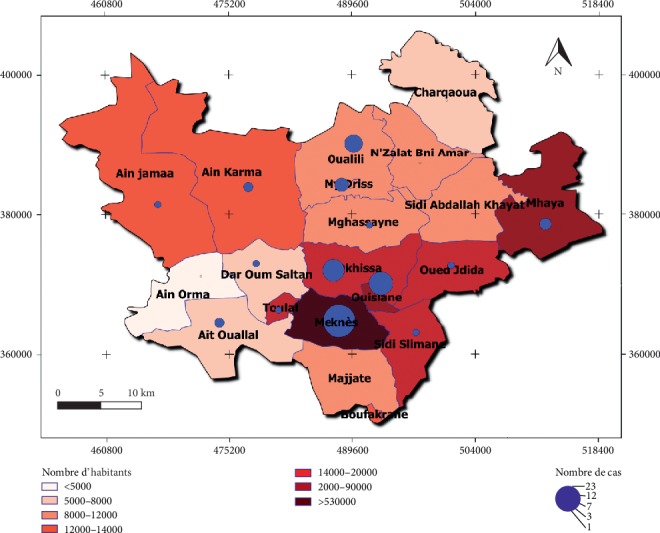
Distribution number of cases of CL and number of population.

The distribution of the CL according to the population at the level of the prefecture of Meknes shows that the Meknes most infested commune (23cas) is characterized by a large overpopulation, which exceeds 530000 inhabitants (Figure 5).

The rural commune Ain Orma Nzalla Bni Amar, Sidi Abdallah Al Khayat, Charkaoua, and Mjjat, which has a smaller number of population, did not register any cases during the study period.

Statistical analysis with the Pearson test showed a strong correlation between the population rate and the number of cases recorded, with a coefficient *R* = 0.849 and *p* ≤ 0.001. Therefore, the distribution of the CL within this prefecture takes into account the number of inhabitants per municipality.

## 4. Discussion

Leishmaniases are notifiable diseases in Morocco. They are classified by WHO as one of the world's leading public health problems [13], with this mind, this study was conducted to determine the impact of urbanization and socioeconomic factors on the distribution of CL in the center of Morocco (prefecture of Meknes).

According to WHO: “Poor housing conditions and peridomestic hygiene (lack of waste management and open sewers) can increase the number of breeding places and resting places for sandflies and their access to humans” [13]. It is for these reasons that we have chosen as risk factors poverty rate, urbanization, and the number of inhabitants per municipality.

The annual revolution of CL showed the endemic nature of the disease in our study area; indeed, the distribution of CL during the study period shows that during the period 2009–2015, the number of cases recorded was low, with the exception of a peak recorded in 2012, which could be explained by the implementation of the Ministry of Health's leishmaniasis control program while using insecticides for the control of leishmaniasis, and by raising awareness among the population.

Insect control was achieved through the introduction of insecticide-treated mosquito nets and the spraying of insecticide by the Infrastructure Services and Provincial Outpatient Acts (SIAAP). This insecticide spraying aimed at interrupting parasitic cycle by destroying one of its links, which is the vector; indeed, with the actions of the struggle put in place by the Moroccan State, the incidence of the CL is in regression. In addition to vector control, the control program aims, on the one hand, to reinforce screening activities, to take early charge of all detected cases, and to undertake appropriate actions to fight against reservoirs; on the other hand, it aims to ensure the training and retraining of the staff of the epidemiological unit. All these actions are at the origin of this decrease in the number of cases.

It should also be noted that the prefecture of Meknes is made up of 20 communes and contains 48 health centers (including 33 urban health centers (CSU) and 15 rural health centers (CSR)) and finally 16 rural clinics, which facilitates the access of the population to diagnosis and treatment.

The increase in cases in 2012 and the permanent existence of the disease could be explained by the insufficiency of the efforts made to fight against this pathology, especially with the increasing density of the population and the propagation of the favorable environments for the multiplication of the vector [9, 14], whereas the fight companions practiced remain untargeted, in absence an in-depth study of the epidemiological profile of the province and also breeding grounds for vectors [15, 16].

The high percentage of cases in the urban environment reveals the strong relationship between CL and urbanization (*r* = 0.49, *p* value = 0.02).

These results could be explained by the increase of the population and the anarchic urbanization generating unhealthy habitats where hygiene conditions are rudimentary. These factors are already reported by Dr. Philippe Desjeux (former Head of Trypanosomiasis and Leishmaniasis Control Programs Division of WHO-Geneva Tropical Disease Control) as a risk factor [17].

In addition, in some cases, the population of rural origin settled in urban areas, and bring back with them domestic animals, they could even carry out livestock farms onsite, which offered suitable media for the multiplication of sandflies [18, 19].

These results are similar to other studies [20–22], who showed that this form of disease began to appear in some periurban and urban sites.

Most of the municipalities affected are urban areas with a low poverty rate: Meknes, Ouislane communes, therefore, poverty does not explain in our case the emergence of the CL (*R* = −0.544 and *p* value = 0.013).

These results are consistent with the study of Desplanques and Piovano [23]. On the other hand, some studies have shown that poverty may increase in some ways the risk of leishmaniasis due to poor hygienic conditions [24, 25]. Moreover, our study highlights that the distribution of leishmaniasis was influenced by the population rate (*R* = 0.849 and *p* ≤ 0.001). Indeed the rural communes Ain Orma Nzalla Bni Amar, Sidi Abdullah Al Khayat, Charkaoui, and Mjjat, which have a smaller number of populations, did not register any cases during the study period. Therefore, the distribution of the CL within this prefecture takes into account the number of inhabitants per municipality.

## 5. Conclusions

In light of these results, we note that the CL exists during all the years of study affecting most of the municipalities with predominance of the urban environment. It also emerges that the poverty of the communes has no impact on the number of cases; on the other hand, the number of inhabitants and the urbanization positively impact the number of cases of leishmaniasis. The results of this research show that the efforts to fight against this disease are still insufficient, hence the need to take into consideration the population and the effect of urbanization in the implementation of surveillance strategies and the fight against this disease.

## Data Availability

The data used in this study are included within the article.
